# Spectral contrast effects are modulated by selective attention in “cocktail party” settings

**DOI:** 10.3758/s13414-019-01824-2

**Published:** 2019-07-23

**Authors:** Hans Rutger Bosker, Matthias J. Sjerps, Eva Reinisch

**Affiliations:** 1 Max Planck Institute for Psycholinguistics, PO Box 310, 6500 AH Nijmegen, The Netherlands; 2 Donders Institute for Brain, Cognition and Behaviour, Radboud University, Nijmegen, The Netherlands; 3 Institute of Phonetics and Speech Processing, Ludwig Maximilian University Munich, Munich, Germany; 4 Institute of General Linguistics, Ludwig Maximilian University Munich, Munich, Germany

**Keywords:** Spectral contrast, Vowel normalization, Selective attention, Stream segregation, “Cocktail party” listening

## Abstract

Speech sounds are perceived relative to spectral properties of surrounding speech. For instance, target words that are ambiguous between /bɪt/ (with low F1) and /bɛt/ (with high F1) are more likely to be perceived as “bet” after a “low F1” sentence, but as “bit” after a “high F1” sentence. However, it is unclear how these spectral contrast effects (SCEs) operate in multi-talker listening conditions. Recently, Feng and Oxenham (*J.Exp.Psychol.-Hum.Percept.Perform. 44*(9), 1447–1457, 2018b) reported that selective attention affected SCEs to a small degree, using two simultaneously presented sentences produced by a single talker. The present study assessed the role of selective attention in more naturalistic “cocktail party” settings, with 200 lexically unique sentences, 20 target words, and different talkers. Results indicate that selective attention to one talker in one ear (while ignoring another talker in the other ear) modulates SCEs in such a way that only the spectral properties of the attended talker influences target perception. However, SCEs were much smaller in multi-talker settings (Experiment 2) than those in single-talker settings (Experiment 1). Therefore, the influence of SCEs on speech comprehension in more naturalistic settings (i.e., with competing talkers) may be smaller than estimated based on studies without competing talkers.

**Electronic supplementary material:**

The online version of this article (10.3758/s13414-019-01824-2) contains supplementary material, which is available to authorized users.

## Introduction

Speech is a highly variable signal: the same word can sound very differently depending on the talker’s gender, vocal tract, mood, and even the room acoustics. One perceptual principle that listeners rely on to deal with part of this variation is spectral contrast. When the spectral content of a given carrier sentence differs from a following target sound, the auditory system perceptually enhances this difference. This is referred to as a spectral contrast effect (or contrast enhancement), with perception of the target being biased away from prominences in the spectrum of the preceding carrier sentence. Spectral contrast effects (SCEs) are typically demonstrated by showing that a lead-in sentence can influence categorization of a following target vowel, consonant, or word. For instance, the perception of a vowel that is spectrally ambiguous (e.g., on an artificially created vowel continuum) between /ɪ/ (with greater energy in the lower range of the first formant, F1; 375–450 Hz) and /ɛ/ (with greater energy in the higher F1 range; 550–625 Hz) is biased towards /ɪ/ when preceded by a carrier sentence with greater energy *above 500 Hz* in the long-term average spectrum (i.e., with a relatively high F1), but towards /ɛ/ when preceded by a carrier sentence with greater energy *below 500 Hz* (i.e., with a relatively low F1; Ladefoged & Broadbent, 1957). The vast majority of studies on SCEs have assessed the role of SCEs in speech comprehension using single-talker listening environments. As a consequence, little is known about how SCEs operate in arguably more natural multi-talker situations with multiple competing speech streams. The present study demonstrates the presence of SCEs with two competing speech streams in “cocktail party” settings. Interestingly, only the spectral properties of the attended stream influence target perception. However, SCEs are sharply reduced in multi-talker listening conditions compared to single-talker settings – irrespective of the spectral characteristics of the competing talker’s speech.

Spectral contrast effects affect a wide range of spectrally cued phonemic contrasts, including vowels (F2 contrast between /ɑ/ vs. /a:/; Bosker, Reinisch, & Sjerps, 2017; Reinisch & Sjerps, 2013; F1 contrast between /ɪ/ vs. /ɛ/; Sjerps, McQueen, & Mitterer, 2013; Stilp & Assgari, 2018), consonants (/b/ vs. /g/; Lotto & Kluender, 1998; /s/ vs. /f/; Sjerps & Reinisch, 2015), lexical tones (Huang & Holt, 2009; Sjerps, Zhang, & Peng, 2018), and even whole words (“Laurel” vs. “Yanny”; Bosker, 2018). Empirical evidence suggests that SCEs are not specific to speech or language, as they are also induced by filtered noise (Watkins & Makin, 1994) and pure tones (Holt, 2005, 2006).

Some have suggested that the context effects described as SCEs involve a form of talker normalization, underlying our ability to resolve variation arising from anatomical vocal tract differences (Ladefoged & Broadbent, 1957). It is suggested to listeners that they construct a representation of the speech patterns of a particular talker (e.g., a cognitive model of the expected vowel space), which serves as a reference frame for the interpretation of subsequent sounds. Thus, it is the talker-specific and speech-specific patterns in a carrier sentence that bias perception of following target sounds. Studies in support of this view have for instance shown that visual cues to talker gender (Johnson, Strand, & D’Imperio, 1999) and explicit instructions about talker gender (Johnson et al., 1999) both induce context effects that are at least qualitatively similar to SCEs.

Others have challenged this view, suggesting that SCEs involve general auditory processes that compute a representation of the average energy across frequencies, like a long-term average spectrum (LTAS). This average spectral representation of a context then serves as a referent for representation for subsequent sounds (Feng & Oxenham, 2018a; Holt & Lotto, 2002; Huang & Holt, 2009; Laing, Liu, Lotto, & Holt, 2012; Lotto & Holt, 2006; Stilp & Assgari, 2018; Watkins, 1991), independent of talker knowledge. That is, exposure to contexts with greater energy *below 500 Hz* results in contrastive enhancement of the frequencies *above 500 Hz* in following ambiguous target vowels, biasing perception of ambiguous /ɪ-ɛ/ vowels towards /ɛ/. Similarly, contexts with greater energy *above 500 Hz* results in contrast enhancement of the frequencies *below 500 Hz* in following targets, resulting in more /ɪ/ responses. This general auditory account is supported by evidence that (speech and non-speech) contexts matched on LTAS produce similar SCEs (Laing et al., 2012), although others have reported differential SCEs for LTAS-matched contexts (Assgari & Stilp, 2015).

Central processing mechanisms have been suggested to contribute at least in part to SCEs. For instance, even though SCEs are strongest when carriers and targets are presented to the same ear, some effects still remain when presented to opposite ears (Feng & Oxenham, 2018b; Holt & Lotto, 2002; Watkins, 1991). Furthermore, SCEs are also observed when carriers and targets are separated by several hundred milliseconds, again suggesting the involvement of more central adaptation mechanisms (Holt, 2005). However, so far neither framework (“talker normalization” vs. “general auditory” accounts) has specified the role of directed attention in SCEs.

The potential modulating influence of attention on SCEs is particularly important when considering multi-talker listening conditions (i.e., listening to an attended talker in the presence of competing speech, known as "cocktail party" settings; McDermott, 2009), where listeners are required to attend to one talker while ignoring others. How do SCEs operate in these arguably more natural, and at the same time much more variable listening conditions? Even though attention is a strong factor in the cortical processing of speech sounds (Kerlin, Shahin, & Miller, 2010; Mattys, Brooks, & Cooke, 2009; Mattys & Wiget, 2011; Mesgarani & Chang, 2012), evidence for attentional modulation of SCEs is rather limited. For instance, Sjerps, McQueen, and Mitterer (2012) demonstrated that SCEs were as strong for participants who, besides categorizing ambiguous /pɪt-pɛt/ target words, were additionally tasked to detect small amplitude dips in carrier sentences (compared to participants who did not perform this secondary task but only categorized the target words). Bosker, Reinisch, and Sjerps (2017) assessed whether increases in cognitive load would modulate SCEs by imposing a secondary task onto participants, using an easy versus a difficult visual search task. During the presentation of manipulated carrier sentences, participants additionally searched for an oddball shape in a small versus large grid of objects. Even though the small versus the large grid manipulation had a large influence on participants’ visual search accuracy, the size of SCEs induced by carrier sentences under low versus high cognitive load conditions did not differ.

The only study, to date, reporting small but significant attentional effects on SCEs is a recent study by Feng and Oxenham (2018b). They examined SCEs in the presence of competing sounds, aiming at distinguishing peripheral from more central context effects. SCEs were assessed by measuring the effect of the spectral properties of two preceding carrier sentences on the categorization of the single target contrast /bɪt/ “bit” versus /bɛt/ “bet”. The first set of experiments used a single carrier sentence and served as a baseline to the second set of experiments. The first set demonstrated that SCEs were present in both ipsilateral and contralateral (i.e., same ear vs. different ear) presentation of carrier + target combinations. Context effects were considerably reduced with contralateral presentation. As such, outcomes of their first set of experiments emphasized the contribution of peripheral mechanisms to SCEs, while at the same time demonstrating that higher-level factors occurring after binaural integration of information also play a role.

The second set of experiments in Feng and Oxenham (2018b) assessed the role of attention in SCEs by presenting listeners with two simultaneously presented sentences (“The last word you hear is” and “You will also hear a sound”), both spoken by the same talker with matched average F0, followed by the target continuum from “bit” to “bet.” Participants were always instructed to attend the sentence “The last word you hear is” and ignore the sentence “You will also hear a sound.” When the two sentences were dichotically presented (i.e., to opposite ears) and the target words either to the attention-ipsilateral ear or the attention-contralateral ear (Experiment 2A), target categorization depended mostly on the ear of presentation – and much less so on attention. That is, if participants were presented with a sentence filtered to emphasize the spectrum of /ɪ/ (“low F1”) on the left and a sentence filtered to emphasize the spectrum of /ɛ/ (“high F1”) on the right, followed by an ambiguous target word on the left, categorization was mostly biased by the spectral properties of the left ipsilateral sentence (i.e., towards /ɛ/) – with only a small modulating effect of whether participants attended left or right. However, when targets were presented diotically to both ears, a more pronounced effect of attention was observed: attending to a “low F1” sentence (and ignoring a “high F1” sentence) biased perception of the diotic target word towards /ɛ/ – irrespective of the ear of presentation of the carrier sentences.

The study by Feng and Oxenham (2018b) is, to our knowledge, the only study to investigate how SCEs operate in the presence of competing sounds. It is also the first to provide some evidence, albeit small, for attentional modulation of SCEs. However, some aspects about that study prevent a straightforward generalization of their findings to more naturally occurring multi-talker (“cocktail party”) settings. First, the same talker was recorded producing both carrier sentences, with matched F0. As such, listeners were presented with the relatively unnatural scenario of a single talker producing two sentences at the same time. More critically, this may have led to an *underestimation* of the modulating effect of attention in “cocktail party” settings, since cognitively segregating sentences from the same talker is more difficult than segregating different talkers (Brungart, 2001). Second, the lexical content of the speech materials was quite restricted (only one attended sentence, one competing sentence, and one target continuum from “bit” to “bet”), which does not reflect more typical conversational settings. Moreover, participants were instructed to always attend one particular sentence – not one particular talker, as one would typically do in “cocktail party” situations. This may have led to *overestimation* of the modulating effect of attention in “cocktail party” settings, since cognitively separating highly predictable sentences is easier than unpredictable sentences (Dai, McQueen, Hagoort, & Kösem, 2017).

Third, only “mismatching” combinations of carrier sentences were tested: when a “low F1” carrier was played in one ear, a “high F1” carrier was played in the other ear (and vice versa). While this maximally distinguishes the two carriers, allowing assessment of the effect of attention, it does not allow examination of the contribution of the ignored carrier to target perception. That is, even if target categorization is biased towards /ɛ/ when attending a “low F1” carrier sentence and ignoring a “high F1” carrier sentence (i.e., following the attended carrier, as reported in Feng & Oxenham, 2018b), how would target categorization change if both attended and ignored carrier sentences had a “low F1”? If target categorization would be even more biased towards /ɛ/ in a trial with two “low F1” carriers (compared to a “low F1” + “high F1” trial), this would indicate that the spectral properties of the ignored carrier sentence still influence target categorization to some degree – despite attentional modulation. In contrast, if target categorization would be comparable irrespective of the spectral properties of the ignored carrier sentence, this would indicate that selective attention is such a strong factor that it completely removes the contribution of the ignored sentence to target perception. Thus, the fact that Feng and Oxenham (2018b) did not include “matching” carrier combinations precludes a more fine-grained understanding of the power of attentional modulation in SCEs.

The present study aimed to assess how SCEs operate in “cocktail party” listening conditions, with typically variable lexical content, different talkers, and various spectral properties of attended and ignored talkers. To achieve this aim, this study built on Feng and Oxenham (2018b), while using lexically diverse carriers and targets, and speech from different talkers. Moreover, the inclusion of both “matching” and “mismatching” carrier combinations served to assess the extent of attentional modulation: can the spectral signature of an unattended competing talker at a “cocktail party” influence perception of an attended talker? That is, does attentional modulation of SCEs mean that the spectral properties of an unattended competing talker influence the perception of an attended talker “only less” or “not at all”?

**Fig. 1:**
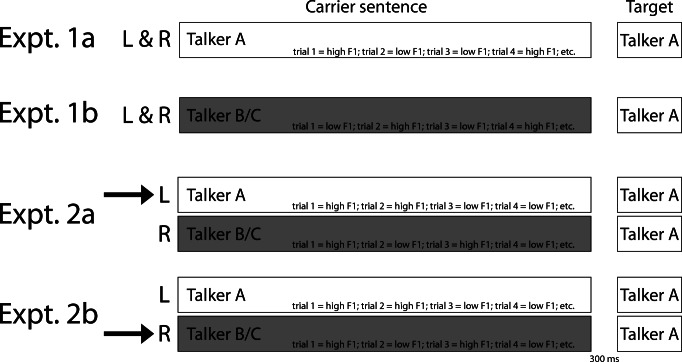
Experimental design. Schematic diagram of the experimental design of the four experiments. Participants were always presented with carrier sentences followed by target words (after a 300-ms silent interval) played over headphones. Target words were always produced by Talker A (white bar), containing manipulated vowels ambiguous between /ɪ/ and /ɛ/ (e.g., *bid* /bɪt/ “pray” – *bed* /bɛt/ “bed”). In Experiment 1a, participants were presented with a single talker producing one carrier sentence at a time (in both ears; always Talker A), with either greater energy in the higher F1 range (HIGH F1) or the lower F1 range (LOW F1; F1 manipulation intermixed across trials). In Experiment 1b, participants were presented with carrier sentences from another talker (either Talker B or C, counter-balanced across participants; gray), resulting in talker-incongruency between carrier and target. In Experiments 2a and 2b, participants were presented with a multi-talker listening situation. They simultaneously heard two different carrier sentences, one in each ear (L/R location counter-balanced across participants). One carrier was always produced by Talker A (talker-congruency with target), the other always by another talker (e.g., Talker B; talker-incongruency with target). Whether the energy in the lower or higher F1 range was enhanced, was fully randomized, resulting in four different possible combinations: two “matching” conditions (Low + Low; High + High), and two “mismatching” conditions (High + Low, Low + High). In Experiment 2a, participants were instructed to attend to Talker A (arrow) and ignore the other talker, co-varying talker-congruency with selective attention. In Experiment 2b, participants listened to the same stimuli, but this time receiving instructions to attend the talker-incongruent carrier sentences (Talker B or C; arrow) and ignore the talker-congruent carrier sentences (Talker A). *Expt* experiment, *L* left audio stream, *R* right audio stream

We performed two experiments. Experiment 1, using single-talker carrier sentences, served as a baseline to Experiment 2, using multi-talker carrier sentences in each trial (i.e., two carriers sentences presented simultaneously). Specifically, inclusion of single-talker Experiment 1 allowed for the comparison of SCEs induced by F1-manipulated carrier sentences in quiet (Experiment 1) versus with a competing talker (Experiment 2). In Experiment 1, two separate groups of Dutch participants listened to combinations of 200 unique carrier sentences and 20 ambiguous target pairs that differed minimally in their word-medial vowels (e.g., /bɪt - bɛt/, /hɪk - hɛk/, /sxɪp - sxɛp/, etc.). Carrier sentences were manipulated to have greater energy in either the lower F1 range (“low F1”; ca. 375–450 Hz) or the higher F1 range (“high F1”; ca. 550–625 Hz). The participants in Experiment 1 heard one carrier sentence followed by a target word. The target words were always from Talker A while the carrier sentence could be either from Talker A (Experiment 1a); or Talker B or C (Experiment 1b; see Fig. 1). Thus, Experiment 1 provides a benchmark for the strength of SCEs when assessing the influence of selective attention in multi-talker settings in Experiment 2.

Based on previous literature on SCEs, hearing a “low F1” carrier sentence before the ambiguous target words should bias target categorization towards /ɛ/, while “high F1” carrier sentences would bias towards /ɪ/. Experiment 1b was included to verify whether speech from a different talker can influence the perception of another talker in the first place. That is, only if we find evidence for SCEs induced by talker-incongruent carrier sentences in Experiment 1b can we attempt to assess whether and how the spectral properties of an unattended competing talker at a “cocktail party” might influence perception of another attended talker in Experiment 2. Previous studies suggest that SCEs occur even when the talker changes between carriers and targets (Assgari & Stilp, 2015; Lotto & Kluender, 1998; Watkins, 1991), although some studies found that talker-incongruency can reduce the effect size of SCEs (Lotto & Kluender, 1998). Therefore, we expect to observe SCEs in Experiment 1a and 1b, although they may be reduced in Experiment 1b.

In multi-talker Experiment 2, participants were presented with two carrier sentences at the same time, one in each ear, followed by one target word (played in both ears; materials drawn from Experiment 1; see Fig. 1). One of the sentences was in the same voice as the target (Talker A), while the other was in a different voice (Talker B or C). The energy in the lower and higher F1 range in the carriers was manipulated within each talker, resulting in four possible combinations: two “matching” conditions in which the spectral content of *both sentences* contained greater energy in the higher F1 range (High + High) or the lower F1 range (Low + Low); and two “mismatching” conditions in which the spectral content in the two sentences was opposed between speakers (High + Low; Low + High). Crucially, half of the participants were instructed to always attend to the various carrier sentences produced by Talker A in one ear and ignore the other (interfering) talker in the other ear (Experiment 2a), while the other half was instructed to attend the various talker-incongruent carrier sentences (i.e., Talker B or C) and ignore the talker-congruent carrier sentences (Talker A; Experiment 2b).

This experimental setup allowed us to test whether selective attention modulates SCEs by presenting participants with a large set of lexically unique sentences and targets, mimicking more typical “cocktail party” settings. The two carrier sentences on a given trial are also produced by two different talkers, assessing whether a *different* competing talker can influence perception of an attended talker (cf. same competing talker in Feng & Oxenham, 2018b). Moreover, fully combining “low F1” and “high F1” carrier sentences (mismatching: Low + High, High + Low; matching: Low + Low; High + High) allows for the assessment of how competing spectral characteristics modulate the effect attended spectral characteristics have on target perception. Does a competing “high F1” carrier lead to fewer /ɛ/ responses than a competing “low F1” carrier? Alternatively, the addition of a competing talker in another ear could also reduce SCEs in general (even if the spectral characteristics of the competing talker are similar to those of the attended talker) as a result of increased attentional load (e.g., greater difficulty segregating the two talkers) and/or reduced reliability of contextual spectral cues (e.g., “relevant” attended spectral characteristics in context are less reliable, hence reducing SCEs). Finally, a comparison across Experiment 2a and 2b will reveal whether the potential modulatory effect of selective attention interacts with talker-congruency. Thus, we aim to assess the contribution of SCEs to speech comprehension in more naturalistic multi-talker situations.

## Experiment 1

### Method

#### Participants

Thirty-two native Dutch participants (24 females, eight males; mean age = 22 years, range = 19–27) with normal hearing were recruited from the Max Planck Institute’s participant pool. We collected data from 16 participants for each individual experiment, which is comparable to earlier studies (Assgari & Stilp, 2015, p. 2015; Bosker et al., 2017; Feng & Oxenham, 2018b; Sjerps & Reinisch, 2015). Participants in all experiments reported in this study gave informed consent as approved by the Ethics Committee of the Social Sciences department of Radboud University (project code: ECSW2014-1003-196). Half of the 32 participants in Experiment 1 took part in Experiment 1a (talker-congruent carriers and targets), the other half in Experiment 1b (talker-incongruent carriers and targets).

#### Materials and design

Two hundred Dutch carrier sentences were constructed, each comprising 20–27 syllables (see Table S2 in Supplementary Materials). All sentences were semantically neutral with regard to the sentence-final target word and did not contain the vowels /ɪ/ or /ɛ/. Twenty Dutch monosyllabic minimal word pairs were selected as targets. The word pairs differed only in their vowel, containing either /ɪ/ or /ɛ/ (e.g., *bid* /bɪt/ “pray” vs. *bed* /bɛt/ “bed”; see Table S1 in Supplementary Materials). The /ɪ-ɛ/ vowel contrast in Dutch is primarily cued by F1 (Adank, Van Hout, & Smits, 2004), with /ɪ/ having a relatively lower F1 (average female F1 in Dutch: 399 Hz) than /ɛ/ (535 Hz; Adank et al., 2004).

**Fig. 2:**
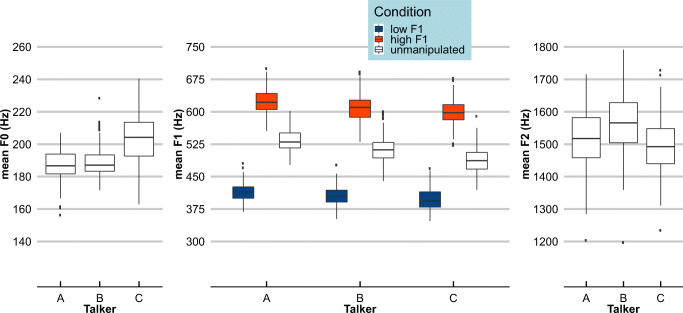
Mean F0, F1, and F2 values for the context sentences from each talker. Boxplots show the mean F0, F1, and F2 values of all context sentences for each talker separately. Measurements were taken from vocalic segments only. F1 was shifted up (orange/light gray) or down (blue/dark gray) by 20% for the “high F1” and “low F1” conditions, respectively

**Fig. 3:**
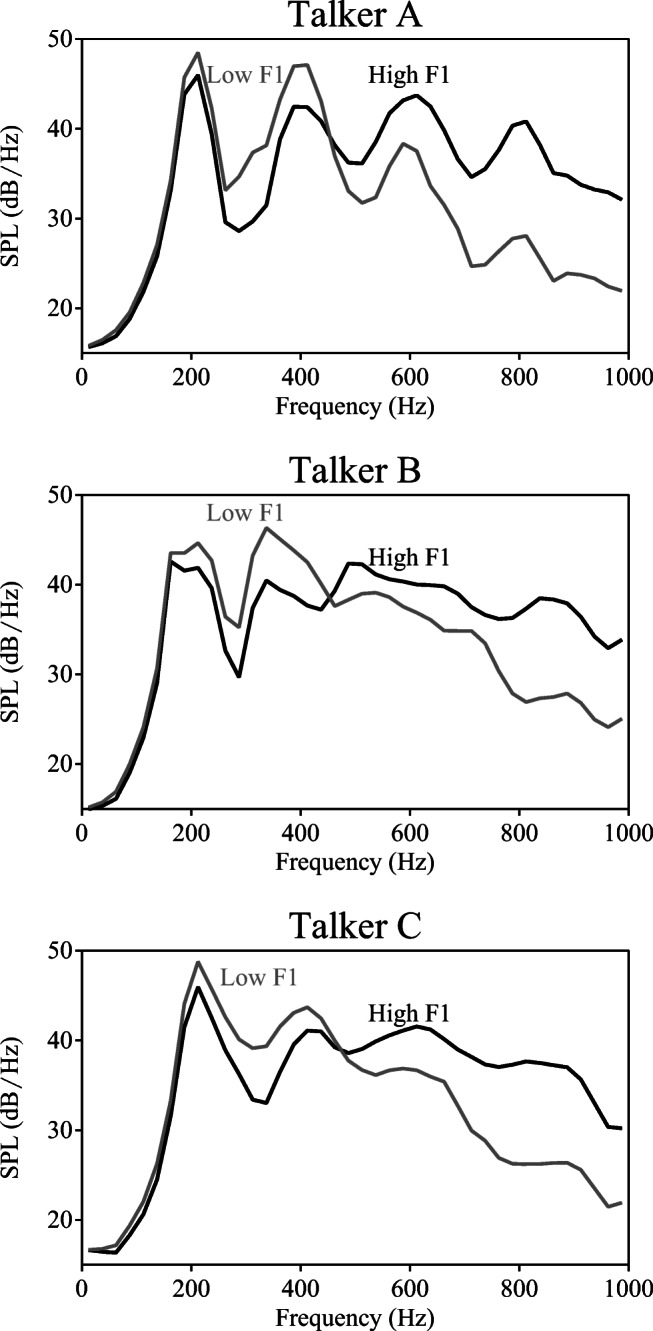
Long-term average spectra (LTASs) of the manipulated carrier sentences in Experiment 1a and 1b. These LTASs show the F1 manipulation in the carrier sentences, with sentences from the “Low F1” conditions containing relatively more energy below ~500 Hz than sentences from the “High F1” conditions (similarly across the three talkers). *SPL* sound pressure level

Three female native speakers of Dutch (referred to as Talkers A, B, and C) were recorded producing all sentences ending in one of the target words. Carrier sentences (i.e., all speech up to target onset) were excised and mean F0, F1 and F2 were calculated using Burg’s LPC method (implemented in Praat; Boersma & Weenink, 2016; cf. Fig. S1 in Supplementary Materials). First, each sentence was set to the mean duration of all sentences that shared the same number of syllables, calculated across all three speakers (using PSOLA in Praat; Boersma & Weenink, 2016). This ensured that sentences with the same number of syllables all had the same length. Secondly, first formant frequencies were manipulated (shifted up and down) using Burg’s LPC method, with the source and filter models estimated automatically from each sentence individually. The first formant track of the filter model of each carrier sentence was increased or decreased by 20%, after which the filter model was recombined with the source model, resulting in a “high F1” with greater energy in the higher F1 range (ca. 550–625 Hz) and a “low F1” version of each carrier sentence with greater energy in the lower F1 range (ca. 375–450 Hz; referred to as the "Praat method" in Feng & Oxenham, 2018b; cf. Winn & Litovsky, 2015). Finally, all carriers were matched in amplitude. Long-term average spectra (LTAS) confirmed that the F1 manipulations had the desired outcomes (see Figs. 2 and 3).

**Fig. 4:**
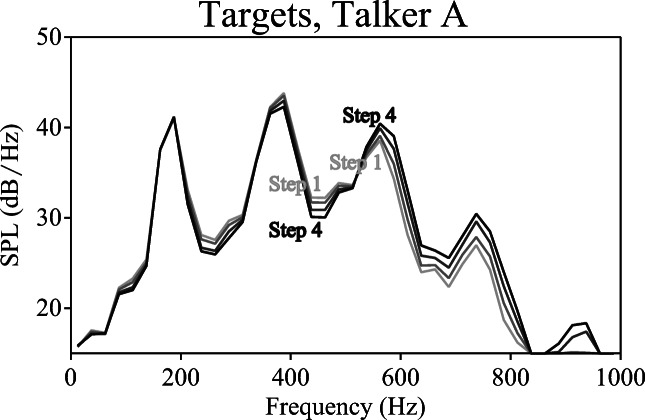
Long-term average spectra (LTASs) of the manipulated target words. These LTASs show the manipulation of the target words on the spectral continuum (only experimental items shown; steps 1–4). The LTAS of step 1 (lightest gray; relatively /ɪ/-like) contains greater energy below ~500 Hz, while the LTAS of step 4 (darkest gray; relatively /ɛ/-like) contains greater energy above ~500 Hz. *SPL* sound pressure level

For the target words, only recordings from Talker A were used (i.e., target words produced by Talker B and C were never used in any of the experiments). Each of the 20 target word pairs was manipulated to create spectral continua of their vowels from /ɪ/ to /ɛ/. For each individual pair, the vowels /ɪ/ and /ɛ/ were identified and first matched in duration and F0 (set to the mean of both) using PSOLA resynthesis in Praat. Then, we used sample-by-sample linear interpolation by mixing the weighted sounds of the pair (9-point continuum; step 1 = 100% /ɪ/ + 0% /ɛ/; step 5 = 50% /ɪ/ + 50% /ɛ/; step 9 = 0% /ɪ/ + 100% /ɛ/; i.e., a step size of 12.5%) to create nine different steps changing in vowel quality. We selected this manipulation method over other possible alternatives (e.g., LPC decomposition), because it resulted in more naturally sounding output and did not require additional item-specific adjustments. These manipulated vowel tokens were then spliced into the consonantal frame from the /ɛ/ member of each pair (i.e., the consonantal frame *b_d* from *bed*). An informal categorization pretest was carried out, using the manipulated target words in isolation (i.e., without a precursor) in order to determine the perceptually ambiguous target range. Based on those outcomes, the same four ambiguous steps on the 9-point continuum (specifically: the second, third, fourth, and fifth steps) were selected for all pairs. Long-term average spectra (LTAS) of these four steps confirmed that the F1 manipulations had the desired outcomes: more /ɪ/-like tokens (e.g., step 1) had greater energy in the lower F1 range (ca. 375–450 Hz), more /ɛ/-like tokens (e.g., step 4) had greater energy in the higher F1 range (ca. 550–625 Hz; see Fig. 4). Moreover, the unambiguous first and last steps on the continuum were selected for use in filler trials (see *Procedure*) to provide participants with a full range of target sounds. These items were used in the main experiments.

#### Procedure

Participants were presented with combinations of carrier sentences and target words over headphones. In Experiment 1a, both the carriers and the targets were produced by Talker A (see Fig. 1). In Experiment 1b, the targets were still produced by Talker A (allowing for a comparison of perceptual categorization across experiments), but the carriers were produced by a different talker (Talker B/C; see Fig. 1). The identity of the talker in the carrier sentence was consistent within but counter-balanced across participants. That is, half of the participants listened to carrier sentences spoken by Talker B, and the other half listened to carriers spoken by Talker C.

The 200 unique carrier sentences were divided into experimental trials (80%, *n* = 160) and filler trials (20%, *n =* 40). Half of the carriers in experimental trials were presented in the “High F1” condition; the other half in the “Low F1” condition. Using a Latin Square design, each participant was presented with both high and low F1 carrier sentences, while avoiding repetition of the same sentence. That is, two stimulus lists were created counter-balancing the F1 of the carrier sentences. These experimental carrier sentences were combined with all targets at the four different ambiguous steps of the spectral continua. Each target sound (20 pairs × 4 steps; *n* = 80) was presented twice: once after a “High F1” carrier sentence and once after a “Low F1” carrier sentence. All target pairs were also presented at the two unambiguous endpoints of the spectral continua – half following a filler carrier with “Low F1” and half following a filler carrier with “High F1”.

Stimulus presentation was controlled by Presentation software (v16.5; Neurobehavioral Systems, Albany, CA, USA). Each trial started with the presentation of a fixation cross. After 500 ms, the carrier sentence was presented, followed by a silent interval of 300 ms, followed by a target word. All speech, that is, carrier sentences and targets, was always presented in both ears. After target offset the fixation cross was replaced by a screen with two response options (i.e., the words of the minimal pair), one on the left, one on the right. The position of response options was counter-balanced across participants. Participants entered their response as to which of the two response options they had heard (*bid* or *bed*, etc.) by pressing the “Z” button on a regular QWERTY computer keyboard for the option on the left, or “M” for the option on the right. After their response or timeout after 4 s, the screen was replaced by an empty screen for 500 ms, after which the next trial was initiated automatically. Participants were given three opportunities to take a short break at a quarter of the experiment, half-way through, and at three-quarters of the experiment. The experiment took approximately 30–40 min to complete.

### Results

All speech stimuli and data from the present study, together with an R analysis script, are available for download (under a CC BY-NC-ND 4.0 license) from: https://osf.io/3n5cv.

**Fig. 5:**
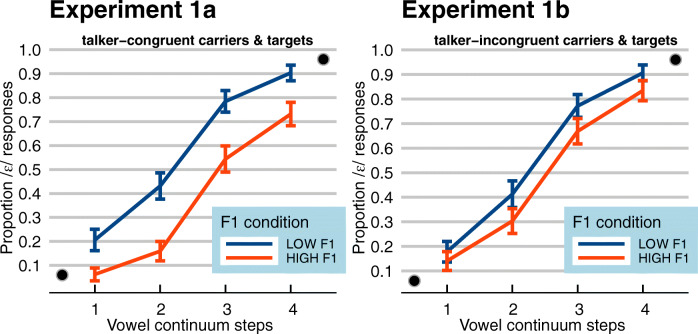
(Color online) Average categorization data of Experiment 1a (single talker; carriers talker-congruent with targets) and Experiment 1b (single talker, carriers talker-incongruent with targets). Data are plotted as the proportion of /ɛ/ responses, with the x-axis indicating steps on the vowel continua, ranging from relatively /ɪ/-like (step 1) to relatively /ɛ/-like (step 4). Blue (dark gray) lines show the “Low F1” carriers, the orange (light gray) lines the “High F1” carriers, indicating that target words preceded by single carrier sentences with greater energy in the lower F1 range bias perception towards /ɛ/. Black dots inside the panels indicate categorization data of unambiguous target steps in filler trials. Error bars enclose 1.96 × SE on either side, that is, the 95% confidence intervals

Trials with missing categorization responses (*n* = 1; < 1%) were excluded from all analyses. Categorization data in filler trials showed that the endpoints of the continua were categorized as intended with close to floor/ceiling performance (0.06 vs. 0.96 proportion of /ɛ/ responses across Experiments 1a and 1b). Categorization data in experimental trials, that is the selected ambiguous steps of the continuum, calculated as the proportion of /ɛ/ responses, *P*(/ɛ/), are presented in Fig. 5. As expected, higher steps on the spectral vowel continuum led listeners to report more /ɛ/ responses (lines have a positive slope). The difference between the orange (light gray) and blue (dark gray) lines indicates an influence of the preceding carrier: carriers with greater energy in the lower F1 range (“Low F1”; blue/dark gray lines) biased perception towards /ɛ/, whereas carriers with greater energy in the higher F1 range (“High F1”; orange/light gray lines) biased perception towards /ɪ/. However, the difference between the two lines across the two panels would seem to be reduced in Experiment 1b compared to Experiment 1a: the overall difference in *P*(/ɛ/) between “Low F1” versus “High F1” was 0.21 in Experiment 1a but 0.08 in Experiment 1b.

We quantified these effects using a generalized linear mixed model (GLMM; Quené & Van den Bergh, 2008) with a logistic linking function as implemented in the lme4 library (version 1.0.5; Bates, Maechler, Bolker, & Walker, 2015) in R (R Development Core Team, 2012). The binomial dependent variable was participants’ categorization of the target in experimental trials as either containing /ɛ/ (e.g., *bed*; coded as 1) or containing /ɪ/ (e.g., *bid*; coded 0). Fixed effects were Continuum Step (continuous predictor; centered and scaled around the mean), Carrier Condition (categorical predictor; deviation coding, with “High F1” coded as -0.5 and “Low F1” as +0.5), Talker-Congruency (categorical predictor; with Experiment 1b mapped onto the intercept), and their interactions. The GLMM included Participant and Target Item as random factors, with by-participant and by-item random slopes for Carrier Condition. More complex random effects structures failed to converge.

This model revealed significant effects of Continuum Step (*β* = 2.039, *SE* = 0.077, *z =* 26.619, *p <* 0.001; higher *P*(/ɛ/) for higher continuum steps) and Carrier Condition (*β* = 0.683, *SE* = 0.155, *z =* 4.413, *p <* 0.001; higher *P*(/ɛ/) for carriers with lower F1). Moreover, an interaction between Carrier Condition and Talker-Congruency (*β* = 1.059, *SE* = 0.219, *z =* 4.837, *p <* 0.001) indicated that the effect of Carrier Condition was more pronounced in Experiment 1a compared to Experiment 1b.

### Discussion

The results of Experiment 1a and 1b showed that our target spectral continua appropriately sampled the perceptual continuum from /ɪ/ (e.g., *bid*) to /ɛ/ (e.g., *bed*). They also demonstrated that carriers with greater energy in the lower F1 range biased target perception to more /ɛ/ responses relative to the same target word preceded by a carrier with greater energy in the higher F1 range (i.e., a shift of 0.21 *P*(/ɛ/)). This replicates earlier spectral contrast findings with similar effect sizes, and serves as a baseline for the following experiments. The results of Experiment 1b additionally showed that SCEs are also induced by F1-manipulated carrier sentences in another voice – albeit to a smaller extent than the talker-congruent carrier sentences in Experiment 1a. As such, it raises the possibility that the spectral properties of an unattended talker may influence the perception of another attended talker in a “cocktail party” setting.

## Experiment 2

Experiment 2 set out to address the question about SCEs in cocktail-party settings. The material was identical to Experiment 1 except that on each trial another, lexically different, carrier sentence produced by another talker (B or C) was played simultaneously to the other ear (see Fig. 1). Crucially, half of the participants were instructed to always selectively attend to Talker A and ignore the other (interfering) talker in the other ear (Experiment 2a), while the other half was instructed to attend the talker-incongruent carrier sentences (i.e., Talker B or C) and ignore the talker-congruent carrier sentences (Talker A; Experiment 2b).

If selective attention modulates spectral contrast effects in speech perception, we would predict that target perception “follows” the F1 of the attended carrier: when attending to a carrier with greater energy in the lower F1 range, one would predict more /ɛ/ responses independent of the spectral characteristics of the to-be-ignored carrier. Comparison across Experiment 2a and 2b will reveal whether this potential modulatory effect of selective attention interacts with talker-congruency.

### Method

#### Participants

Thirty-two native Dutch participants (25 females, seven males; mean age = 23 years, range = 19–35) with normal hearing that had not participated in Experiment 1a or 1b were recruited from the Max Planck Institute’s participant pool.

#### Materials and design

The stimulus lists from Experiment 1, counter-balancing F1 energy across carrier sentences, formed the basis of the lists in Experiment 2. The carrier sentences from Talker A were paired with other lexically different carrier sentences from Talker B or C such that talker identity was counter-balanced across participants (i.e., each participant only ever heard either talker B or C as competing talker). Paired sentences were to selected to have the same number of syllables. Half of Talker A’s “High F1” carrier sentences were paired with other (matching) “High F1” carrier sentences, the other half paired with other (mismatching) “Low F1” carrier sentences. Similarly, half of Talker A’s “Low F1” carrier sentences was paired with other (matching) “Low F1” carrier sentences, the other half paired with other (mismatching) “High F1” carrier sentences.

**Fig. 6:**
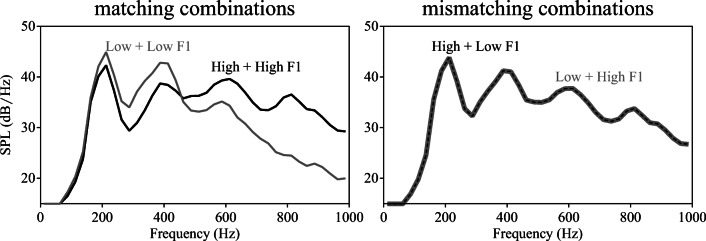
Long-term average spectra (LTASs) of the carrier pairs in Experiment 2. In the left panel, combining two carriers with F1 shifted down (Low + Low) resulted in greater energy in the lower F1 range, while combining two carriers with F1 shifted up (High + High) resulted in greater energy in the higher F1 range. In the right panel, combining two mismatching carriers (High + Low, Low + High) resulted in highly similar LTAS patterns. *SPL* sound pressure level

As a result, participants heard four different combinations of carriers: High + High, High + Low, Low + Low, Low + High (see Fig. 1). The LTASs of the four carrier combinations (see Fig. 6) confirmed that combining two carriers with F1 shifted down (Low + Low) resulted in greater energy in the lower F1 range in the LTAS compared to combining two carriers with F1 shifted up (High + High). In contrast, combining two mismatching carriers (High + Low, Low + High) resulted in highly similar LTAS patterns. Due to the matching described in the Method of Experiment 1, both members of each pair had an equal duration and intensity (0 dB target-to-masker ratio). Due to the counter-balancing described above, all targets appeared in all possible conditions an equal number of times.

#### Procedure

The procedure of Experiment 2 was similar to the procedure used in Experiment 1, except that an additional “to-be-ignored” carrier sentence was played on each trial. Half of the participants was instructed to selectively attend the carrier sentences by Talker A (Experiment 2a), while the other half was instructed to selectively attend the carrier sentences by Talker B/C (Experiment 2b). The allocation of talkers to ears was consistent within a given participant but counter-balanced across participants. This meant that participants selectively attended to only one ear + talker combination throughout an experimental session. Carrier Condition was varied within participants on a trial-by-trial basis; that is, in one trial the attended talker had greater energy in the higher L1 range, on another the attended talker had greater energy in the lower F1 range. Target words were always played in both ears. Despite the unnatural transition from carrier sentences (each played in one ear) to target words (played in both ears), we selected this method for reasons of experimental control. Specifically, it provides equal opportunity for either carrier sentence to influence the following target word, since the target word matched both sentences in ear-of-presentation. If only peripheral factors influenced target categorization, then we would expect both sentences to equally influence target perception. If, instead, attention modulates spectral contrast effects in speech perception, we would predict that target perception would follow the F1 of the attended carrier.

To assess how successful participants were in selectively attending to the to-be-attended talker, participants were at times presented with prompts to type out the words from the last attended carrier sentence. These prompts were presented after half of the filler trials (*n =* 20 out of 200 trials in total) after participants had provided a categorization response. We explicitly instructed participants to enter all words they remembered from the attended sentence and to guess if necessary. Finally, we also asked participants to fill out a debriefing questionnaire after participating in the experiment, enquiring about the perceived difficulty of the attentional task, how successful they were in attending to the to-be-attended talker and ignoring the other talker, and potential strategies.

### Results

Trials with missing categorization responses (*n* = 8; < 1%) were excluded from all analyses. Before turning to the categorization data, we briefly discuss participants’ reported words in the sentence prompts to assess whether the attention manipulation had worked.

#### Selective attention

The mean proportion of words reported from the *attended* sentence was 0.65 (*SD* = 0.28) in Experiment 2a and 0.62 (*SD* = 0.31) in Experiment 2b. The mean proportion of words reported from the *unattended* sentence was < 0.01 (*SD* = 0.03) in both Experiment 2a and Experiment 2b. Only in eight trials (< 0.01%; 4 in Experiment 2a, 4 in Experiment 2b) did participants ever report more words from the unattended sentence than from the attended sentence. These numbers were comparable in trials with matching and mismatching conditions.

Responses on the debriefing questionnaire revealed a similar pattern. Participants reported that selectively attending to one talker and ignoring the other was demanding but doable. At times, the attentional focus on the attended talker was lost due to fatigue or curiosity about the speech from the other talker, but most participants reported that it was relatively easy to restore selective attention. Strategies involved, for instance, concentrating harder, closing one’s eyes, looking in the direction of the attended sound, etc. Some participants commented on the F1 manipulation, usually in terms of “height” or “pitch,” but no participant reported strategic use of the F1 manipulation in perceptual categorization.

#### Categorization data

**Fig. 7:**
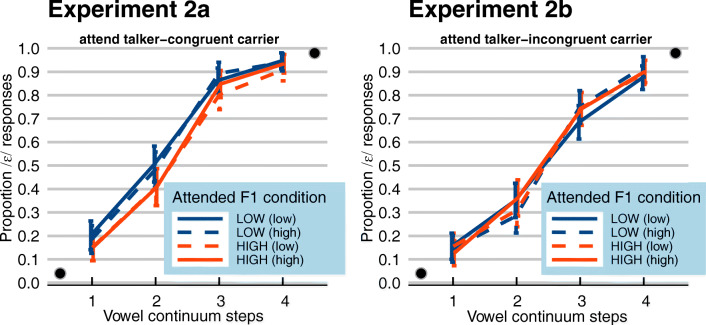
(Color online) Average categorization data of Experiment 2a (selective attention to *talker-congruent* carrier sentences) and Experiment 2b (selective attention to *talker-incongruent* carrier sentences). Data are plotted as the proportion of /ɛ/ responses, with the x-axis indicating steps on the vowel continua, ranging from relatively /ɪ/-like (step 1) to relatively /ɛ/-like (step 4). The various lines represent the various carrier conditions (attended F1 in capitals, ignored F1 in parentheses). The solid lines show the matching conditions (HIGH (high); LOW (low)), the dashed lines show the mismatching conditions (HIGH (low); LOW (high)). Black dots inside the panels indicate categorization data of unambiguous target steps in filler trials. Error bars enclose 1.96 × SE on either side, that is, the 95% confidence intervals

Categorization data in filler trials again showed that the endpoints of the continua were categorized as intended with close to floor/ceiling performance (0.04 vs. 0.98 proportion of /ɛ/ responses across Experiments 2a and 2b). Categorization data in experimental trials, calculated as the proportion of /ɛ/ responses, *P*(/ɛ/), are presented in Fig. 7. Carrier Condition is coded as “ATTENDED F1 (ignored F1)”, with the *attended* carrier in capitals and the *unattended* carrier in lower case in parentheses. Again, as in Experiment 1, higher steps on the vowel continua led listeners to report more /ɛ/ responses. In addition, in Experiment 2a (left panel) attending to a carrier sentence with greater energy in the lower F1 range (while ignoring another carrier; blue/dark gray lines) seems to induce more /ɛ/ responses, relative to attending to a carrier sentence with greater energy in the higher F1 range (orange/light gray lines). This effect appears to be independent from the spectral characteristics of the unattended carrier (solid vs. dashed lines). However, in Experiment 2b (right panel), there does not seem to be any consistent difference between the various carrier conditions.

A GLMM tested for fixed effects of Continuum Step, Attended Condition (categorical predictor; deviation coding, with “high attended F1” coded as -0.5 and “low attended F1” as +0.5), and Experiment (categorical predictor; with Experiment 2a mapped onto the intercept), and all interactions. By-item random intercepts and slopes for all fixed factors were included, as well as by-participant random intercepts and slopes for Continuum Step and Attended Condition. Note that a comparison of this model to a more complex model including the additional predictor Match (comparing trials with matching vs. mismatching conditions) did not reveal a significant difference (*χ*^*2*^(8) = 9.974, *p =* 0.267). Therefore, we pooled across trials with matching and mismatching conditions in the data from Experiment 2.

This GLMM revealed a significant effect of Continuum Step (*β* = 2.664, *SE* = 0.228, *z =* 11.679, *p <* 0.001; higher *P*(/ɛ/) with higher continuum steps). Also, an effect of Attended Condition was observed (*β* = 0.565, *SE* = 0.232, *z =* 2.432, *p =* 0.015), indicating that attended carriers with greater energy in the lower F1 range received more /ɛ/ responses than carriers with greater energy in the higher F1 range. However, an interaction between Attended Condition and Experiment (*β* = -0.680, *SE* = 0.325, *z =* -2.092, *p =* 0.036) revealed that the effect of Attended Condition was only observed in Experiment 2a; not in Experiment 2b (i.e., the *β* estimate of Attended Condition: 0.565; *minus* 0.680 even gives a slight negative effect).

In order to compare the effect of Carrier Condition across all experiments (i.e., Experiment 1a, 1b, 2a, and 2b), we tested whether there was statistical evidence for interactions between the effect of Attended Condition and Experiment. We combined the data from all four experiments and entered these into an omnibus GLMM, containing fixed effects for Continuum Step, Attended Condition (categorical predictor; deviation coding, with ‘high attended F1’ coded as -0.5 and ‘low attended F1’ as +0.5), Experiment (categorical predictor; with Experiment 2a mapped onto the intercept), and all interactions. By-item random slopes for Continuum Step, Attended Condition, and Experiment were included, as well as by-participant random slopes for Continuum Step and Attended Condition.

This GLMM revealed significant effects of Continuum Step and Attended Condition corroborating the results already reported in Experiment 2a, since Experiment 2a was mapped onto the intercept. Most importantly, the model revealed a significant interaction between Attended Condition and the contrast between Experiment 2a and Experiment 1a (*β* = 1.329, *SE* = 0.285, *z =* 4.664, *p <* 0.001). This indicates that the effect of Attended Condition was significantly larger in Experiment 1a compared to that in Experiment 2a. That is, the addition of a “to-be-ignored” carrier sentence produced by another talker significantly reduced (but did not remove) the spectral contrast effect. A significant interaction between Attended Condition and the contrast between Experiment 2a and Experiment 2b (*β* = -0.669, *SE* = 0.282, *z =* -2.372, *p =* 0.018) indicated that the effect of Attended Condition was significantly reduced in Experiment 2b compared to that in Experiment 2a (cf. the effect of Experiment in the GLMM on Experiments 2a and 2b above). No difference was observed between the effect of Attended Condition in Experiment 2a versus 1b (*β* = 0.195, *SE* = 0.280, *z =* 0.695, *p =* 0.487).

A mathematically equivalent GLMM, this time mapping Experiment 2b onto the intercept, additionally revealed significant interactions between Attended Condition and the contrast between Experiment 2b, mapped onto the intercept, and Experiment 1a (*β* = 1.990, *SE* = 0.281, *z =* 7.080, *p <* 0.001). This indicates that the effect of Attended Condition was significantly larger in Experiment 1a compared to that in Experiment 2b. Also, an interaction between Attended Condition and the contrast between Experiment 1b and Experiment 2b was found (*β* = 0.863, *SE* = 0.277, *z =* 3.116, *p =* 0.001), indicating that the effect of Attended Condition was significantly larger in Experiment 1b compared to that in Experiment 2b. That is, the addition of a (unattended) talker-congruent carrier sentence significantly reduced, and even removed, the spectral contrast effect induced by talker-incongruent carrier sentences.

### Discussion

The findings from Experiment 2 provided several insights. First, target perception was only influenced by the spectral characteristics of the attended talker-congruent carrier, independent of the spectral characteristics of the ignored talker-incongruent carrier. Second, the spectral contrast effect induced by the attended talker-congruent carriers in Experiment 2a was considerably reduced compared to Experiment 1a. That is, the addition of another ‘irrelevant’ talker during the carrier sentence significantly reduced the spectral contrast effect induced by the attended talker. Third, no spectral contrast effect was observed in any condition in Experiment 2b. In fact, even though a small spectral contrast effect was observed in Experiment 1b with single talker-incongruent carriers, this effect was further reduced and even removed by additionally presenting another ‘to-be-ignored’ talker. This suggests that spectral contrast is not only sensitive to talker-congruency with the target, but also to the presence of interfering talkers.

## General discussion

This study assessed the influence and potential limits of low-level speech perception processes (here spectral contrast effects; SCEs) that listeners have been shown to use in dealing with variability in the speech signal due to different talker characteristics. We specifically asked what the contribution of directed attention is to SCEs in multiple-speaker listening environments (“cocktail party” settings). We built on the only previous empirical piece of evidence for attentional modulation of SCE (Feng & Oxenham, 2018b), assessing whether listeners, when presented with two talkers at the same time, are influenced by the spectral properties of the attended talker only, or also by the spectral characteristics of the other competing talker.

Spectral contrast effects were successfully established in both single-talker Experiments 1a and 1b: target words ambiguous in their word-medial vowel (e.g., /bɪt/ vs. /bɛt/) were more likely to be perceived as containing the vowel /ɛ/ (with greater energy in the higher F1 range, ca. 550–625 Hz) if preceded by a carrier sentence with greater energy in the lower F1 range (ca. 375–450 Hz). Conversely, if the same target word was preceded by a carrier sentence with greater energy in the higher F1 range (ca. 550–625 Hz), target perception was biased towards hearing /ɪ/. When the carrier sentences were produced by a different talker than the one who produced the targets (Experiment 1b), SCEs were also observed – albeit significantly reduced compared to hearing the same talker throughout (Experiment 1a).

This reduced effect size is in line with the reduced SCEs in Lotto and Kluender (1998), but conflicts with Watkins (1991) and Assgari and Stilp (2015) who showed comparable effect sizes for SCEs induced by talker-congruent and talker-incongruent contexts. Reduced SCEs for talker-incongruent contexts are also intriguing considering the two accounts about the cognitive mechanisms underlying SCEs. A “talker normalization” account would predict no SCEs at all when ambiguous targets are preceded by talker-incongruent carrier sentences, because the talker-specific patterns in the carrier sentence would not apply to the target speech from another talker. A “general auditory” account, on the other hand, would predict SCEs of similar magnitude for talker-congruent and talker-incongruent contexts, manipulated with similar F1 shifts. Therefore, the present reduction of SCEs when targets are preceded by talker-incongruent contexts could indicate the operation of two concurrent types of mechanisms, one driven by general auditory contrast and another driven by talker-specific normalization processes. However, since the present study was not designed to investigate the cognitive mechanisms that underlie the reduction of SCEs in talker-incongruent speech materials, we can only speculate about this issue. Future studies may assess the extent to which general auditory and talker-specific normalization mechanisms are interrelated (e.g., operating in parallel or serially). Crucially for our current purposes, the outcomes of Experiment 1b showed that SCEs are sensitive to the spectral properties of another talker, raising the possibility that an unattended talker might influence the perception of another attended talker in a “cocktail party” situation.

Experiment 2 investigated whether SCEs induced by talker-incongruent carriers are still present in “cocktail party” settings, when there are two spatially-segregated carrier sentences, produced by different talkers, one in each ear. In Experiment 2a, participants were instructed to always attend the talker-congruent carriers and ignore the talker-incongruent carrier sentences (see Fig. 1), most resembling a typical “cocktail party” setting. The use of a large set of lexically diverse carrier sentences ensured that participants could not use the lexical content of carrier sentences to maintain selective attention, reflecting somewhat more naturalistic “cocktail party” settings (cf. Feng & Oxenham, 2018b). Results indicated that target perception was influenced by the spectral properties of the attended talker only: when the attended talker had greater energy in the lower F1 range, target perception was biased towards /ɛ/, independent of the spectral properties of the speech of the competing talker. Specifically, in Experiment 2a, target categorization did not differ between trials with a to-be-ignored competing talker with “low F1” versus “high F1”; that is, whether the spectral energy profile in the F1 range of the ignored talker matched or mismatched the spectral energy profile in the F1 range of the attended talker. This extends our understanding of attentional modulation of SCEs as first reported in Feng and Oxenham (2018b). It suggests that selective attention in “cocktail party” settings can suppress the contextual influence from a competing talker’s speech completely: only the spectral characteristics of attended talkers exert a contrastive effect on following perception.

This finding is arguably an efficient characteristic of speech comprehension in multi-talker listening environments, attenuating potentially interfering influences from competing speech sources. This is in stark contrast with recent findings concerning a related contextual phenomenon involving *temporal* contrast effects – also known as rate normalization (Bosker & Ghitza, 2018; Bosker & Reinisch, 2017; Kaufeld, Ravenschlag, Meyer, Martin, & Bosker, in press; Maslowski, Meyer, and Bosker, 2019a, b). That is, duration cues on target speech sounds are perceived relative to the surrounding speech rate: a word with a reduced unstressed syllable (e.g., “-um” in “forum”) is perceived as “shorter” (“form”) in a slow context, but “longer” (“forum”) in a fast context (Baese-Berk, Dilley, Henry, Vinke, & Banzina, 2018; Dilley & Pitt, 2010). However, when such ambiguous target words are perceived in the context of two carrier sentences, one slow and the other fast (like in Experiment 2), target categorization is *not* influenced by selective attention to one or the other sentence (Bosker, Sjerps, & Reinisch, 2018). That is, target perception is not different when attending the fast versus the slow sentence, in contrast to the outcomes about *spectral* contrast effects in Experiment 2a. This could indicate that differential cognitive mechanisms underlie the two seemingly analogous normalization processes (spectral normalization as studied here vs. rate normalization), as indeed suggested by recent neurobiological studies (Kösem et al., 2018; Sjerps, Fox, Johnson, & Chang, 2019).

Note, however, that although the spectral characteristics of the competing talker did not influence SCEs, the sheer presence of competing speech did influence the magnitude of SCEs. That is, Experiment 1a – with large SCEs – and Experiment 2a – with reduced SCEs – only differed in the absence or presence of a competing talker. This indicates that the contribution of SCEs to naturalistic speech comprehension, as previously assessed using single-talker materials, may be overestimated when it comes to multi-talker environments.

Note that, first of all, the reduced effect size in Experiment 2a relative to Experiment 1a is unlikely to be driven by the fact that the attended talker was presented to only one ear in Experiment 2a, but to two ears in Experiment 1a. Indeed, Feng and Oxenham (2018b) convincingly demonstrated that ear of presentation is a major factor in SCEs. However, even the SCEs induced by “matching” conditions (High + High vs. Low + Low) in Experiment 2a can be seen to be considerably smaller (in left panel of Fig. 7) than the SCEs in Experiment 1a (in left panel of Fig. 5), despite both ears receiving comparable spectral stimulation (cf. Figs. 3 and 6). Hence, this reduced effect size must be driven by higher-level factors.

For instance, it could be argued that the reduced effect size of SCEs in multi-talker situations was driven by an increase in attentional load in perceptual processing of two talkers at the same time. However, previous studies have directly manipulated cognitive load during the presentation of carrier sentences and have not found modulating effects thereof. As introduced above, Bosker et al. (2017) did not find differences in the size of SCEs under low versus high cognitive load, as manipulated by means of easy versus difficult visual search tasks. While in the case of Bosker et al. it could be argued that the imposed cognitive load affected a different perceptual modality (i.e., vision), Sjerps et al. (2012) also observed SCEs of similar effect sizes when participants were additionally tasked to detect small amplitude dips in carrier sentences. Note, however, that despite the auditory nature of the secondary task in Sjerps et al., in both studies the tasks were non-linguistic (visual search and auditory amplitude dip detection). In contrast, the present Experiment 2a also differed from Experiment 1a in its linguistic attentional load, since participants additionally had to remember (and occasionally recall) the words of the attended sentence. Assessment of whether and how linguistic and non-linguistic attentional loads modulate SCEs would be an interesting avenue for follow-up research, especially considering the “general auditory” view that SCEs involve *domain-general* normalization processes (Bosker et al., 2017; Holt & Lotto, 2002; Laing et al., 2012; Reinisch & Sjerps, 2013; Sjerps & Reinisch, 2015).

Alternatively, the reduction of SCEs in multi-talker situations could have been due to the reduced reliability of the spectral cues in the attended carrier sentences. Perhaps the spectral cues to the lower versus higher F1 in the attended carrier sentences was masked by the competing cues in the unattended carrier sentence. This could have made the contextual cues less accessible to listeners and/or less reliable, in turn reducing their effect on subsequent target perception. Future work may attempt to test whether the reliability of contextual cues modulates SCEs by, for instance, comparing speech-in-quiet versus speech-in-noise.

The outcomes of Experiment 2b provided further support for the conclusions introduced above. First, even though the acoustic stimuli in Experiment 2b were identical to those in Experiment 2a, different categorization patterns were observed depending on which talker participants were instructed to attend. This corroborates the finding that SCEs are modulated by selective attention. Second, while SCEs were observed for single talker-incongruent carrier sentences in Experiment 1b (albeit reduced), no evidence for SCEs was found when the same talker-incongruent sentences were presented together with another competing talker. This is in line with the reduction of SCEs in Experiment 2a versus Experiment 1a, thus providing additional empirical support for the present conclusions.

Together, the current experiments speak to the debate about what cognitive and perceptual mechanisms underlie SCEs: “general auditory” spectral contrast (Laing et al., 2012) or “talker normalization” (Ladefoged & Broadbent, 1957)? As discussed above, the most parsimonious account of the effects observed here draws from both views, since the reduction of SCEs by talker-incongruent contexts supports “talker normalization,” while the sheer presence of SCEs (albeit reduced) for talker-incongruent contexts points to a “general auditory” talker-independent interpretation. In any case, both accounts will have to be updated to incorporate modulation by selective attention since neither of the accounts have been applied to multi-talker communicative settings before. In the case of the “general auditory” account, this may be achieved through enhanced coding of spectral speech properties by selective attention. Research has shown that auditory attention can strongly modulate cortical responses to speech sounds (Mesgarani & Chang, 2012), which would presumably then also affect the average spectral representation of contextual speech thought to induce SCEs. In the case of the “talker normalization” account, switching one’s attention between two talkers would presumably involve the activation of different representations of talker-specific speech patterns, hence inducing differential SCEs.

### Conclusion

This study compared spectral contrast effects in single-talker versus multi-talker listening conditions. In “cocktail party” settings, where listeners are attending one talker while ignoring another, perception of the attended talker is contrastively influenced by the preceding spectral properties of the attended talker only. In fact, we find evidence that the spectral properties of an unattended talker can be completely ignored by selectively attending the attended talker. However, spectral contrast effects in multi-talker environments are reduced relative to hearing the same attended talker in isolation. Thus, we show that the contribution of spectral contrast effects to more naturalistic listening situations may be modest, highlighting the need for studying the processes involved in speech perception in their natural habitat.

## Electronic supplementary material

ESM 1 (DOCX 39 kb)
