# Supplementary material for: Spectral contrast effects are modulated by selective attention in “cocktail party” settings
Source: Atten Percept Psychophys. 2019 Jul 23;82(3):1318–32. doi: 10.3758/s13414-019-01824-2 (PMC7303055; doi:10.3758/s13414-019-01824-2)
Supplement: Supplementary file 1 — (DOCX 39 kb) [file 13414_2019_1824_MOESM1_ESM.docx]

**Supplemental Material**

***Spectral contrast effects are modulated by selective attention in ‘cocktail party’ settings***

**Hans Rutger Bosker, Matthias Sjerps, and Eva Reinisch**

Content: Tables S1-S2.

Note that all speech stimuli and data from the present study, together with an R analysis script, are available for download (under a CC BY-NC-ND 4.0 license) from: <https://osf.io/3n5cv>.

Table S1. List of target minimal pairs (*N* = 20) with IPA transcriptions and English translations.

|  | member with /ɪ/ | member with /ɛ/ |
| --- | --- | --- |
| 1 | *bid* /bɪt/ “pray” | *bed* /bɛt/ “bed” |
| 2 | *bik* /bɪk/ “chop” | *bek* /bɛk/ “beak” |
| 3 | *bil* /bɪl/ “buttock” | *bel* /bɛl/ “bell” |
| 4 | *dik* /dɪk/ “fat” | *dek* /dɛk/ “deck” |
| 5 | *hik* /hɪk/ “hiccup” | *hek* /hɛk/ “fence” |
| 6 | *hip* /hɪp/ “hip/trendy” | *heb* /hɛp/ “have” |
| 7 | *lig* /lɪx/ “lie down” | *leg* /lɛx/ “lay down” |
| 8 | *pil* /pɪl/ “pill” | *pel* /pɛl/ “peel” |
| 9 | *pit* /pɪt/ “stone (of a fruit)” | *pet* /pɛt/ “cap” |
| 10 | *rib* /rɪp/ “rib” | *rap* /rɛp/ “rap” |
| 11 | *schip* /sxɪp/ “ship” | *schep* /sxɛp/ “shovel” |
| 12 | *spil* /spɪl/ “pivot” | *spel* /spɛl/ “game” |
| 13 | *stik* /stɪk/ “suffocate” | *stek* /stɛk/ “site” |
| 14 | *stip* /stɪp/ “dot” | *step* /stɛp/ “step” |
| 15 | *strik* /strɪk/ “bow” | *strek* /strɛk/ “stretch” |
| 16 | *wig* /wɪx/ “wedge” | *weg* /wɛx/ “road” or “gone” |
| 17 | *wind* /wɪnt/ “wind” | *went* /wɛnt/ “get used to” |
| 18 | *wit* /wɪt/ “white” | *wed* /wɛt/ “bet” |
| 19 | *wrik* /vrɪk/ “pry” | *vrek* /vrɛk/ “scrooge” |
| 20 | *zich* /zɪx/ “themselves” | *zeg* /zɛx/ “say” |

Table S2. List of context sentences (*N* = 200) with number of syllables (σ) and English paraphrases.

|  | Dutch context sentence | σ | English (literal) paraphrase |
| --- | --- | --- | --- |
| 1 | De muziek op de koptelefoon van haar broer hapert steeds op de uithaal van het woordje | 20 | The music on the headphones of her brother stutters continually on the sustained note of the word |
| 2 | Door het afbreken van de dakpannen op de schuur zag de bouwvakker opeens het woordje | 20 | Because of the breaking off of the roof tiles on the shed saw the construction worker suddenly the word |
| 3 | De spion volgde de verdachte toen deze een afspraak had voor het huis van het woordje | 20 | The spy followed the suspect when he had a meeting in front of the house of the word |
| 4 | Vanaf volgend jaar staat op alle tenues van de lokale basketbalclub het woordje | 20 | From next year stands on all uniforms of the local basketball club the word |
| 5 | De jongens hielden een competitie over wie het langst kan praten over het woordje | 20 | The boys held a competition about who could speak the longest about the word |
| 6 | Toen de vrouw de telefoon opnam, hoorde ze na tien seconden alleen het woordje | 20 | When the woman the phone took, heard she after ten seconds just the word |
| 7 | Deze problematische jonge vrouw fluisterde naar iedere man op straat het woordje | 20 | This problematic young woman whispered to every man on street the word |
| 8 | De zusjes zongen nu al urenlang een uitermate storend lied over het woordje | 20 | The sisters sung now already hours long an extremely disturbing song about the word |
| 9 | Toen de juf de toets van een scholier controleerde op fouten, markeerde ze het woordje | 20 | When the teacher the test of the pupil checked for mistakes, marked she the word |
| 10 | De schoonzus sprak voortdurend over haar beroep als brandweervrouw, maar nooit over het woordje | 20 | The sister-in-law spoke continually about her profession as firefighter, but never about the word |
| 11 | Het leukste cadeau wat jullie hadden gegeven was de ballade over het woordje | 20 | The nicest present what you had given was the ballad about the word |
| 12 | De straatmuzikanten laten het publiek hard lachen door een optreden van het woordje | 20 | The street musicians let the public hard laugh with an appearance of the word |
| 13 | Tot moeders grote frustratie bleef de baby maar huilen na het horen van het woordje | 20 | To mother's greatest frustration continued the baby just crying after the hearing of the word |
| 14 | De stotterende jongen kreeg vooral veel opgaven voor het uitspreken van het woordje | 20 | The stuttering boy got mostly many assignments for the pronouncing of the word |
| 15 | Het was kiezen tussen twee kwaden, of je zweeg een poos of je schreeuwde juist hard het woordje | 20 | It was choosing between two evils, either you were silent a while or you just screamed hard the word |
| 16 | Nadat de bal vlak langs het goal vloog, schreeuwde alle tegenstanders opgelucht het woordje | 20 | After the ball close along the goal flied, screamed all opponents relieved the word |
| 17 | Voor het toernooi moet nog veel geregeld worden, vooral voor de spandoeken van het woordje | 20 | For the tournament a lot must still be arranged, especially for the banners of the word |
| 18 | Door het donkere bos lopen de bruine beren zachtjes te brommen over het woordje | 20 | Through the dark forest walk the brown bears quietly to growl about the word |
| 19 | De slapende slangen snakten 's ochtends nadat ze uitgerust waren zeer naar het woordje | 20 | The sleeping snakes craved in the morning when they rested were strongly to the word |
| 20 | Door een storend geluid konden de toeschouwers niet meer goed de speech horen over het woordje | 20 | Because of a disturbing sound could the spectators not anymore good the speech hear about the word |
| 21 | Het kost de analfabeet moeite om te lezen, behalve als het gaat om het woordje | 20 | It costs the illiterate difficulty to read except if it is about the word |
| 22 | De overgebleven noedels van de noedelsoep vormden geheel onbedoeld het woordje | 20 | The leftover noodles of the noodle soup formed entirely unintentionally the word |
| 23 | Toen de redactrice 's ochtends vroeg de krant open sloeg, viel haar oog meteen op het woordje | 20 | When the editor in the morning early the newspaper open hit, fell her eye immediately on the word |
| 24 | De maniakken die haar achtervolgden bleven staan toen ze hoorden over het woordje | 20 | The maniacs that her followed stayed standing when they heard about the word |
| 25 | Toen de huisgenoten een afspraak hadden gemaakt, riep de jongste bewoner het woordje | 20 | When the housemates an agreement had made, yelled the youngest resident the word |
| 26 | De hooggeleerde mevrouw uitte na de lange afspraak haar wantrouwen voor het woordje | 20 | The highly learned woman expressed after the long meeting her distrust for the word |
| 27 | De mannen van de lokale voetbalclub konden nog steeds niet luisteren naar het woordje | 20 | The men of the local football club could yet still not listen to the word |
| 28 | Het eerste woordje dat de peuter had gesproken op de peuterspeelzaal was het woordje | 20 | The first word that the toddler had spoken in the playgroup was the word |
| 29 | De hele familie lag helaas jaren overhoop door een ruzie over het woordje | 20 | The whole family laid unfortunately years in a mess because of a fight about the word |
| 30 | Toen de vrouw vanochtend naar de koeienstallen liep, hoorde ze daar onverwachts het woordje | 20 | When the woman this morning to the cowshed walked, heard she there unexpectedly the word |
| 31 | De coach probeerde de teamgenoten aan te sporen door het uitroepen van het woordje | 20 | The coach tried the teammates on to spur by the exclamation of the word |
| 32 | Aan de overkant klonk vanochtend volgens de stoere jongens meerdere malen het woordje | 21 | On the opposite side sounded this morning according to the tough boys multiple times the word |
| 33 | Omdat de bladblazers continu lawaai maakten, klaagden de scholieren over het woordje | 21 | Because the leaf blowers continually noise made, complained the students about the word |
| 34 | Het typeert de jonge filosoof om struinend of sloffend te piekeren over het woordje | 21 | It characterizes the young philosopher to rummaging or shuffling to brood about the word |
| 35 | De vrouw was een boek aan het bestuderen waardoor ze alleen nog maar oog had voor het woordje | 21 | The woman was studying a book because of which she only just had eye for the word |
| 36 | Een paar eeuwen geleden was het een gewoonte dat je niet mocht zeuren over het woordje | 21 | A few centuries ago was it a custom that you not may whine about the word |
| 37 | Toen de scooter tegen de auto botste, schreeuwde de autobestuurder woedend het woordje | 21 | When the scooter against the car crashed, screamed the driver furiously the word |
| 38 | De onzekere vrouw stottert niet veel meer, behalve als ze woorden uitspreekt als het woordje | 21 | The insecure woman stuttered not much anymore, except if she words pronounced like the word |
| 39 | De koeien op het grasland keken allemaal geschrokken op na het geluid van het woordje | 21 | The cows on the grassland looked all frightened upwards after the sound of the word |
| 40 | De mooie paardebloemen die speciaal voor haar verjaardag waren geplant vormden het woordje | 21 | The beautiful dandelions that specially for her birthday were planted formed the word |
| 41 | Nadat de kleuter haar knuffel had verloren fluisterde ze tegen haar moeder het woordje | 21 | After the pre-school child her plushy had lost whispered she to her mother the word |
| 42 | Het opvoeden kost veel moeite, zeker wanneer de koters weer eens zeuren over het woordje | 21 | The raising costs much effort, especially when the youngsters again once whine about the word |
| 43 | Na uren weet ze nog steeds niet het goede antwoord op de vraag, dus antwoordt ze maar het woordje | 21 | After hours knows she yet still not the correct answer to the question, so answers she just the word |
| 44 | Vanwege de grote natuurramp was op de markt een somber standbeeld geplaatst voor het woordje | 21 | Due to the great natural disaster was on the market a gloomy statue placed for the word |
| 45 | Vandaag heeft haar moeder maar liefst drie uur lang over de telefoon gepraat over het woordje | 21 | Today has her mother as much as three hours long over the phone talked about the word |
| 46 | De vrouwen konden hun ogen niet geloven toen de mannen abrupt wezen op het woordje | 21 | The women could their eyes not believe when the men abruptly pointed to the word |
| 47 | Toen de psycholoog aan de vrouw vroeg om over haar dromen te praten, noemde ze het woordje | 21 | When de psychologist to the woman asked to about her dreams to speak, mentioned she the word |
| 48 | Op de grote markt stond vandaag een verwarde, dakloze man die iets riep over het woordje | 21 | On the large market stood today a confused, homeless man that something yelled about the word |
| 49 | Toen de supporters de arena verlieten, riepen ze om ons te waarschuwen het woordje | 21 | When the supporters the arena left, yelled they to us to warn about the word |
| 50 | Gedurende de race riep de coach van het roeiteam vanaf de kant naar de stuurman het woordje | 21 | During the race yelled the coach of the rowing team from the side to the coxswain the word |
| 51 | Alle losgeslagen paarden langs de straat liepen terug naar hun stal waarop stond het woordje | 21 | All escaped horses along the street walked back to their stable on which stood the word |
| 52 | Na de slapeloze nachten bezocht de vrouw een dokter die haar waarschuwde voor het woordje | 21 | After the sleepless nights visited the woman a doctor who her warned for the word |
| 53 | De vakantieplannen van de grote vriendengroep moesten veranderd worden door het woordje | 21 | The holiday plans of the large circle of friends had to be changed by the word |
| 54 | De onlangs ontsnapte valkparkieten konden terug vliegen naar hun kooi ondanks het woordje | 21 | The recently escaped cockatiels could back fly to their cage despite the word |
| 55 | De onhandelbare tiener schreeuwde zojuist luidkeels naar haar arme grootmoeder het woordje | 21 | The unmanageable teenager screamed just now loudly to her poor grandmother the word |
| 56 | De moeder was zeer verbaasd want op het karton van de halfvolle yoghurt ontbrak het woordje | 21 | The mother was very surprised because on the cardboard of the semi-skimmed yoghurt lacked the word |
| 57 | De twee politiemannen gebruikten gedurende de klopjacht als codewoord het woordje | 21 | The two policemen used during the round-up as code word the word |
| 58 | De man hoorde de groep jonge vrouwen achter het warenhuis constant klagen over het woordje | 22 | The man heard the group young women behind the department store constantly complain about the word |
| 59 | De kat van de buurvrouw die spoorloos verdwenen leek, was gevonden door het roepen van het woordje | 22 | The cat of the neighbor that traceless disappeared seemed, was found by the yelling of the word |
| 60 | Na maandenlang puzzelen bleek het juiste antwoord van de moeilijke kruiswoordpuzzel het woordje | 22 | After months long puzzling proved the correct answer of the difficult crossword puzzle the word |
| 61 | De gedurfde tiener won de beruchte competitie vanwege haar foto van het woordje | 22 | The daring teenager won the notorious competition by her photograph of the word |
| 62 | De deelnemers drukten allemaal op de rode knoppen, want de presentator riep het woordje | 22 | The participants pressed all on the red buttons, because the presenter yelled the word |
| 63 | Alle auto's op de straat moesten aan de kant gaan, want de ambulance vertoonde het woordje | 22 | All cars on the street had to go to the side, because the ambulance showed the word |
| 64 | De door de warmte opgestegen lucht toont op de zandvlakte van grote afstand het woordje | 22 | The by the warmth risen air showed on the sand plain from great distance the word |
| 65 | De vrouw heeft totaal niet geslapen, want ze lag de hele nacht te piekeren over het woordje | 22 | The woman has totally not slept, because she laid the whole night to worry about the word |
| 66 | Veel toeschouwers verlieten de zaal toen de cabaretier een verhaal begon over het woordje | 22 | Many spectators left the hall when the comedian a story started about the word |
| 67 | Na het poetsen van alle schoenen zal de duizendpoot tevreden gaan dromen over het woordje | 22 | After the polishing of all shoes will the centipede satisfyingly go dreaming about the word |
| 68 | De spreker die een groot publiek toesprak, kreeg plots een lachbui na een reactie over het woordje | 22 | The speaker who a great audience addressed, got suddenly a fit of laughter after a response about the word |
| 69 | Alle jongens van de eerste klas waren geschrokken van de krantenkoppen over het woordje | 22 | All boys of the first class were frightened by the headlines about the word |
| 70 | Achteraf gezien kon de ambtenaar onmogelijk weten wat zo dom was aan het woordje | 22 | Afterwards seen could the public servant impossibly know what so stupid was about the word |
| 71 | De vastgeketende neusapen sloegen helemaal op hol toen ze hoorden over het woordje | 22 | The chained proboscis monkeys ran completely amuck when they heard about the word |
| 72 | De officiele naam voor deze bijzondere gebakjes van de markt bevat het woordje | 22 | The official name for these special pastries from the market contain the word |
| 73 | Op de huishoudbeurs proberen meubelzaken klanten te lokken door het roepen van het woordje | 22 | On the home exhibition try furniture businesses customers to tempt by the yelling of the word |
| 74 | De doorgaans sombere collega kon haar lach niet onderdrukken toen ze luisterde naar het woordje | 23 | The generally gloomy colleague could her laugh not suppress when she listened to the word |
| 75 | De ambtenaar durfde niet langer meer rapporten te typen na het foutief typen van het woordje | 23 | The public servant dared no longer anymore reports to type after the incorrect typing of the word |
| 76 | Deze zwoele zomerse nachten maakten ongemakkelijke gevoelens los over het woordje | 23 | These sultry summery nights made uneasy feelings loose about the word |
| 77 | Om de verborgen poort te openen raak je de uitstekende steen aan zacht fluisterend het woordje | 23 | In order to open the hidden gate, touch you the sticking out stone softly, whispering the word |
| 78 | Om de antwoorden van de toets te kunnen onthouden, schreven de scholieren op hun arm het woordje | 23 | To the answers of the test to can remember, wrote the students on their arm the word |
| 79 | De scholieren hadden veel moeite om een rapport van duizend woorden te typen over het woordje | 23 | The students had much difficulty to a report of thousand words to type about the word |
| 80 | Om tot de studie te worden toegelaten moet de scholier een opdracht maken over het woordje | 23 | In order to get accepted to the study, must the student an assignment make about the word |
| 81 | Toen de papieren onderwater gehouden waren, ontstond door de druk van het water het woordje | 23 | When the papers under water held were, originated by the pressure of the water the word |
| 82 | De vrouw die nog nooit wat gelezen had was zeer enthousiast over een boek dat gaat over het woordje | 23 | The woman who yet never something read had was very enthusiastic about a book that goes about the word |
| 83 | Het koppel dat morgen zou gaan trouwen, blaast de bruiloft af vanwege een ruzie over het woordje | 23 | The couple that tomorrow would go marry, blow the wedding off due to a fight about the word |
| 84 | De regisseur vond het vreselijk moeilijk om een musical te maken gebaseerd op het woordje | 23 | The director found it horribly difficult to a musical to make based on the word |
| 85 | De auteur van de beroemde kookboeken vond het moeilijk een synoniem te kiezen voor het woordje | 23 | The author of the famous cookbooks found it difficult a synonym to choose for the word |
| 86 | De stoere man van de motorclub heeft onlangs een rustgevend luisterboek gekocht over het woordje | 23 | The tough man from the motor club has recently a calming audiobook bought about the word |
| 87 | Toen de dominostenen na maandenlang opbouwen opeens omvielen, schreeuwde ze woest het woordje | 23 | When the domino stones after months-long building up suddenly fell over, screamed she savagely the word |
| 88 | De agrarische markt had voor deze vreselijke situatie nog nooit gehoord van het woordje | 23 | The agricultural market had before this terrible situation yet never heard about the word |
| 89 | De kunstenaars leken tevreden over het onlangs geopende museum over het woordje | 23 | The artists seemed content about the recently opened museum about the word |
| 90 | Op tweede paasdag zongen de tenoren vol vertrouwen een hartverscheurend lied over het woordje | 24 | On second Easter day sung the tenors full of confidence a heartbreaking song about the word |
| 91 | De acteur lag de hele nacht te tobben over wat de media vandaag schreven over het woordje | 24 | The actor laid the whole night to worry about what the media today wrote about the word |
| 92 | Voor een sollicitatie moest de vrouw van een afstand een stuk voorlezen over het woordje | 24 | For an application had to the woman from a distance a piece read aloud about the word |
| 93 | De collega's waren vandaag zeer druk vanwege het ophangen van alle posters over het woordje | 24 | The colleagues were today very busy due to the hanging up of all posters about the word |
| 94 | Het was te betreuren dat de voetballers gedurende de rust niet mochten praten over het woordje | 24 | It was to regret that the football players during the rest not were allowed to talk about the word |
| 95 | De groep vrouwen die uitgelaten de zaal hadden betreden, bleven aldoor gieren over het woordje | 24 | The group women that elated the hall had entered, kept continually shrieking about the word |
| 96 | De deelneemster van het tv-programma was plots gespannen omdat ze steeds niet kon komen op het woordje | 25 | The participant of the television program was suddenly tensed because she continually could not retrieve the word |
| 97 | De onderzoeker vond dat alle kandidaten onderzocht moesten worden op het gebruik van het woordje | 25 | The researcher found that all candidates investigated must get on the use of the word |
| 98 | De computer was kapot gegaan nadat de leraar een artikel wou kopieren over het woordje | 25 | The computer was broken gone after the teacher an article wanted copy about the word |
| 99 | Gedurende zakelijke afspraken debatteren de collega's veel over het gebruik van het woordje | 26 | During commercial meetings debate the colleagues much about the use of the word |
| 100 | Bepaalde organisaties beschouwen dialogen als ongepast als de sprekers praten over het woordje | 27 | Certain organizations considered dialogues as unfit if the speakers talk about the word |
| 101 | De lieve juffrouw sprak vandaag weer over een jongen die vaak moest huilen door het woordje | 20 | The sweet teacher spoke today again about a boy who often had to cry because of the word |
| 102 | Door het afkoelen van de aarde bevroor de sneeuw maar verscheen op de aardkorst het woordje | 20 | Because of the cooling off of the earth froze the snow but appeared on the earth's crust the word |
| 103 | De jongens hoorden het groepje oude dames op school steeds weer praten over het woordje | 20 | The boys heard the group old ladies in school once again talking about the word |
| 104 | De partner van de vrouw heeft haar verlaten omdat ze constant zeurde over het woordje | 20 | The partner of the wife has her left because she constantly whined about the word |
| 105 | De artiest riep gedurende de show die compleet uit de hand liep geregeld het woordje | 20 | The artist shouted during the show that completely out of control went regularly the word |
| 106 | De sporter riep luidkeels voordat de halve marathon begon naar het publiek het woordje | 20 | The sportsman shouted loudly before the half marathon started to the audience the word |
| 107 | Toen de dochter haar dure schoenen verloor, schreeuwde de moeder woedend naar haar het woordje | 20 | When the daughter her expensive shoes lost, yelled the mother furious to her the word |
| 108 | Vanochtend vond de beroemde acteur een oud boek onder een kussen over het woordje | 20 | This morning found the famous actor an old book under a pillow about the word |
| 109 | Op een zonovergoten ochtend vormde de schaduw op de muur van de schuur het woordje | 20 | On a sun-drenched morning formed the shadow on the wall of the shed the word |
| 110 | De oude vader van haar vriend wordt meestal zeer lusteloos als het gaat over het woordje | 20 | The old father of her friend becomes mostly very lethargic when it goes about the word |
| 111 | Als het weer hard gaat sneeuwen moet iedereen een grote sneeuwpop vorm maken van het woordje | 20 | When it again hard goes snowing must everyone a big snowman form make of the word |
| 112 | Als de jongens samen komen om bier te zuipen luisteren ze standaard naar het woordje | 20 | When the guys get together to beer drink, listen they standardly to the word |
| 113 | Toen het uitdelen van de vla te lang duurde, mompelden alle bejaarden het woordje | 20 | When the distributing of the custard too long lasted, mumbled all the elderly the word |
| 114 | Nadat alle cadeaus aan haar waren gegeven, sprak de vrouw als bedankje het woordje | 20 | After all the presents to her were given, spoke the lady as a thank you the word |
| 115 | Als je ooit hulp behoeft, aarzel niet om die te vragen door het gebruiken van het woordje | 20 | If you ever help need, hesitate not to ask by using the word |
| 116 | De bevlogen kapster aan de jachthaven uitte haar grote zorgen over het woordje | 20 | The enthusiastic hair dresser at the yacht-basin expressed her big concerns about the word |
| 117 | De woeste radicalen kwamen samen op de markt om te klagen over het woordje | 20 | The furious radicals came together at the market to complain about the word |
| 118 | Op vakantie had de familie mooie verhalen verzonnen over het woordje | 20 | On holiday had the family beautiful stories made up about the word |
| 119 | De hoge gebouwen hadden grote oranje uithangborden waarop stond het woordje | 20 | The high buildings had big orange billboards on which stood the word |
| 120 | De sluwe zakkenrollers op de rommelmarkt weten alle details over het woordje | 20 | The sly pickpockets at the jumble know all details about the word |
| 121 | Haar ouders waren zeer bezorgd nadat hun dochter een artikel schreef over het woordje | 20 | Her parents were very concerned after their daughter an article wrote about the word |
| 122 | De politieman gebruikte een blauw potlood om de doos te voorzien van het woordje | 20 | The policeman used a blue pencil to the box provide with the word |
| 123 | De buitenlandse producten geven de markt nieuwe mogelijkheden voor het woordje | 20 | The foreign products give the market new possibilities for the word |
| 124 | Als de zusjes gaan shoppen, kopen ze alleen maar modieuze kleren van het woordje | 20 | When the sisters go shopping, buy they only fashionable clothes from the word |
| 125 | Door hardop te praten komt ze weer op het problematische detail over het woordje | 20 | By talking out loud, retrieves she again the problematic detail about the word |
| 126 | De zakenman die verdacht wordt van fraude durft niet langer te praten over het woordje | 20 | The business man who suspected is of fraud, dares no longer to talk about the word |
| 127 | De oude man die gestrand was op het onbewoonde gebied schreef op het strand het woordje | 20 | The old man who stranded had on the uninhabited area wrote on the beach the word |
| 128 | De vrouw heeft slapeloze nachten door al het gepieker over de aard van het woordje | 20 | The woman had sleepless nights because of all the brooding about the gist of the word |
| 129 | Nu de bladeren van de bomen vallen, komt de fase van het wachten op het woordje | 20 | Now the leaves of the trees are falling, comes the phase of the waiting on the word |
| 130 | Toen de computer weer eens vastliep, hoorde je alleen nog maar over en weer het woordje | 20 | When the computer again froze, hear you only back and forth the word |
| 131 | Haar goede vriend kreeg afgelopen zomer een brievenbundel cadeau over het woordje | 20 | Her good friend received last summer a letter bundle for free about the word |
| 132 | Toen de politievrouw de crimineel te pakken kreeg, riep ze triomfantelijk het woordje | 21 | When the policewoman the criminal caught, shouted triumphantly the word |
| 133 | Deze achterbakse matrozen riepen zomaar naar de gewonde skateboarder het woordje | 21 | The sly seamen shouted for no reason to the wounded skateboarder the word |
| 134 | Het hoort niet dat de grote mannen de onschuldige vrouwen zo bang maken voor het woordje | 21 | It ought not that the big men the innocent women so afraid make of the word |
| 135 | Naast het bord waar alle koopjes voor vandaag opstaan, hangt het leuke briefje over het woordje | 21 | Next to the board where all the bargains of today stand, hangs the nice note about the word |
| 136 | Op het verlaten strand probeerde de bange man een groot kampvuur te maken van het woordje | 21 | On the deserted beach tried the scared man a big camp fire to make from the word |
| 137 | De drukke peuter die een huilbui heeft over haar verdwenen speelgoed, heeft troost aan het woordje | 21 | The active toddler who a crying fit has about her vanished toys, finds comfort in the word |
| 138 | Geheel onbedoeld vormden de gehaktballen van haar grootmoeder's tomatensoep het woordje | 21 | Completely unintentional formed the meatballs from her grandmother's tomato soup the word |
| 139 | Op de gekleurde sjaal die de puber heeft gekregen staat door haar oma gehaakt het woordje | 21 | On the colored scarf that the teenager has received stands by her grandma crocheted the word |
| 140 | Alle collega's worden vandaag op kantoor ondervraagd over hun gebruik van het woordje | 21 | All colleagues must today at the office be questioned about their use of the word |
| 141 | Na afloop van het daguitje roddelden de clubleden alleen nog maar over het woordje | 21 | After the daytrip gossiped the club members solely about the word |
| 142 | De bewoners van het dorpje waren zeer ontdaan door het plotse schandaal over het woordje | 21 | The citizens of the village were very upset by the sudden scandal about the word |
| 143 | De opdracht voor het vak Frans was om een rapport van vier kantjes te maken over het woordje | 21 | The assignment for the course French was to a report of four pages make about the word |
| 144 | Door het sleutelgat zag de ongehoorzame puber haar ouders spreken over het woordje | 21 | Through the key hole saw the disobedient teenager her parents talk about the word |
| 145 | Na een lange avond vol avontuur begon de jongen te ratelen over het woordje | 21 | After a long evening full adventures started the boy to rattle about the word |
| 146 | Achter de balie van de klantenservice zong de juffrouw een bijzonder lied over het woordje | 21 | Behind the counter of the customer service sang the lady a special song about the word |
| 147 | De grapjas van de lagere klas verzon de meest hilarische grappen over het woordje | 21 | The joker from junior high made up the most hilarious jokes about the word |
| 148 | Vele jonge handelaren gaven aan dat ze niet goed konden luisteren naar het woordje | 21 | Many young traders pointed out that they not good could listen to the word |
| 149 | Als gevolg van de aanhoudende roddels, gaf de burgermeester een toespraak over het woordje | 21 | As consequence of the continuous gossip, gave the mayor a speech about the word |
| 150 | Toen de ouders van huis waren, kliederde de ondeugende peuter op de muur het woordje | 21 | When the parents from home were, messed the naughty toddler on the wall the word |
| 151 | De vrouw liet na de moeilijke rekentoets niets los over haar gedachten over het woordje | 21 | The woman let after the difficult math test nothing loose about her thoughts about the word |
| 152 | De heerser van het grauwe kasteel heeft een verbod gegeven op het spreken van het woordje | 21 | The ruler of the grimy castle has a prohibition given on the speaking of the word |
| 153 | De uitermate vertroetelde olifanten gehoorzamen alleen nog maar aan het woordje | 21 | The extremely pampered elephants obey only just but to the word |
| 154 | Het geruis van de bomen klonk als een fluisterende peuter die klaagde over het woordje | 21 | The rustling of the trees sounded as a whispering toddler who complains about the word |
| 155 | De jonge vrouw had een brief gestuurd van duizend woorden, maar kreeg alleen als antwoord het woordje | 21 | The young woman has a letter sent of thousand words, but received only as answer the word |
| 156 | De poedel sprong door een brandende hoepel naar de waterbak op commando van het woordje | 21 | The poodle jumped through a burning hoop to the through on command of the word |
| 157 | Nadat de kleuter uit de boom viel, had ze veel krassen op haar been die leken op het woordje | 21 | After the preschooler out the tree fell, had she many scratches on her leg that looked like the word |
| 158 | Op het spandoek dat ze hadden gemaakt voor het kampioenschap stond onder andere het woordje | 22 | On the banner that they had made for the championship stood among others the word |
| 159 | Na het trainen kon de verlegen jongen het woord paddestoel beter uitspreken dan het woordje | 22 | After the training could the shy boy the word mushroom better pronounce than the word |
| 160 | De zeer begaafde tovenaar gaf weer een demonstratie van een toverspreuk over het woordje | 22 | The very gifted wizard gave again a demonstration of a spell about the word |
| 161 | Het lezen van verhalen kan als zeer vermoeiend worden gezien als het gaat over het woordje | 22 | The reading of stories can as very tiring be seen if it is about the word |
| 162 | Op het eeuwenoude altaar stond de priester vol overgave te preken over het woordje | 22 | On the age-old altar stood the priest full commitment to preach about the word |
| 163 | Bovenop de heuveltop vormen de kromgebogen takken van een eenzame boom het woordje | 22 | On top of the hill top formed the crooked branches of the lonely tree the word |
| 164 | De auteur van het beroemde boek beweerde dat ze alle roem te danken had aan het woordje | 22 | The author of the famous book claimed that she all fame to thank had to the word |
| 165 | Door het niet nakomen van de gemaakte beloften ontstaat grote woede over het woordje | 22 | Because of the not complying with the made promises started large anger about the word |
| 166 | Door het nieuwe rooster zullen meerdere vakken veranderen, onder andere het woordje | 22 | Because of the new schedule will multiple courses change, among others the word |
| 167 | De rode scheve ruitjes op het te korte rokje vormden samen het patroon van het woordje | 22 | The red slanting plaids on the too short skirt formed together the pattern of the word |
| 168 | De sluwe vos vermoedde dat de verwaande kraai die een stuk kaas vast had zong over het woordje | 22 | The sly fox suspected that the arrogant crow that a piece cheese fast held sung about the word |
| 169 | Afhankelijk van de uitkomst zal de pastoor besluiten of de speech door zal gaan over het woordje | 22 | Dependent of the outcome will the pastor decide if the speech will go through about the word |
| 170 | De brand heeft alle gewassen verwoest, want al wat overbleef was het woordje | 22 | The fire has all vegetation destroyed, because all that remained was the word |
| 171 | De nietjes van de nietmachine waren op, waardoor ze alle blaadjes los had van het woordje | 22 | The staples of the staples were gone, through which she all papers loose had of the word |
| 172 | Toen de boze juf uitzocht wie de school te vroeg had verlaten, riep ze op luide toon het woordje | 22 | When the angry teacher figured out who the school too early had left, yelled she on loud tone the word |
| 173 | Op de verjaardag van haar oma was de taart versierd door chocolade vlokken met het woordje | 22 | On the birthday of her grandma was the cake decorated by chocolate flakes with the word |
| 174 | Toen de dieven de supermarkt verlieten, schreeuwde de bestolen vrouw achter de kassa het woordje | 23 | When the thieves the supermarket left, screamed the robbed woman behind the cash register the word |
| 175 | Toen de toverkol op haar bezemsteel kwam aanvliegen, riep de tovenaar een spreuk over het woordje | 23 | When the sorceress on her broomstick came flying, yelled the wizard a spell about the word |
| 176 | Als je helemaal niet weet wat je moet kiezen, raadt? ze aan om dan toch maar te kiezen voor het woordje | 23 | If you completely not know what you must choose, advises she to then nevertheless just to choose for the word |
| 177 | De zwarte wolken die door de zware voorjaarsstorm worden voortgeblazen vormen samen het woordje | 23 | The black clouds that by the heavy springtime storm were blown forward form together the word |
| 178 | De hond van de buren luistert super goed, omdat ze gaat rollen op het commando van het woordje | 23 | The dog of the neighbors listens very well, because she starts rolling on the command of the word |
| 179 | Toen de passagiers nieuws zochten over hun aankomstdatum, verscheen plots op het grote bord het woordje | 23 | When the passengers news sought about their arrival date, appeared suddenly on the large sign the word |
| 180 | De speech van de premier die voor de vierde keer opnieuw verkozen was stopte abrupt op het woordje | 23 | The speech of the prime minister who for the fourth time again elected was stopped abruptly on the word |
| 181 | Gedurende het maandelijkse uitje praatte de man constant over liedjes over het woordje | 23 | During the monthly outing talked the man constantly about songs about the word |
| 182 | Om de geblokkeerde deur te openen moet je zachtjes een sprookje fluisteren over het woordje | 23 | To the blocked door to open must you softly a fairytale whisper about the word |
| 183 | De woordvoerder liet weten dat de politicus onderzoek doet naar de zaken rondom het woordje | 23 | The spokesperson let know that the politician investigation does into the matters around the word |
| 184 | Omdat de paarden van de manege waren ontsnapt, zong de staljongen een lied over het woordje | 23 | Because the horses of the stables had escaped, sung the stable boy a song about the word |
| 185 | Voordat het karretje van de achtbaan begon te bewegen schreeuwde de bange vrouw al het woordje | 23 | Before the cart of the rollercoaster begun to move screamed the frightened woman already the word |
| 186 | Naderhand hoorden de betrokken pubers over de gruwelijke geruchten rondom het woordje | 23 | Afterwards heard the involved adolescent about the gruesome rumors around the word |
| 187 | Toen de gasten hun eten hadden verorberd, fluisterde de jongen tegen de ober het woordje | 23 | When the guests their food had consumed, whispered the boy to the waited the word |
| 188 | Gezien de situatie waren alle bewoners van de streek toegesproken over het woordje | 23 | Given the situation were all inhabitants of the region addressed about the word |
| 189 | Achter de afgebakende gebieden zaten de politiemannen te lachen om het woordje | 23 | Behind the marked out areas sat the policemen to laugh about the word |
| 190 | Nadat ze goed had gekeken naar de grote gevaren die op de loer lagen, riep ze plots het woordje | 24 | After she well had looked to the large dangers that on the lurk laid, yelled she suddenly the word |
| 191 | De vrouw las van de week zonder emotie te tonen een hartverscheurend versje voor over het woordje | 24 | The woman read past the week without emotion to show a heartbreaking verse aloud about the word |
| 192 | De verhalen-?voorlezer van de stad las vandaag op de markt een spannend verhaal voor over het woordje | 24 | The storyteller of the city read today on the market an exciting story aloud about the word |
| 193 | Gedurende de avond worden zware buien verwacht boven het land door de orkaan van het woordje | 24 | During the evening were heavy showers expected above the land by the hurricane of the word |
| 194 | Zodra de bruiloft was afgelopen liep de bruidegom tegen de bruid te praten over het woordje | 24 | As soon as the wedding had ended walked the groom to the bride to talk about the word |
| 195 | De druktemakers waren volgens velen te wreed gedurende de demonstraties over het woordje | 24 | The troublemakers were according to many too cruel during the demonstrations about the word |
| 196 | De ongemotiveerde jongen moest van de dokter dagelijks spreektaakjes oefenen over het woordje | 25 | The unmotivated boy had to from the doctor daily speech tasks practice about the word |
| 197 | De verwaarloosde kat van de overburen komt niet om een beetje water bedelen maar om het woordje | 25 | The neglected cat from the opposite neighbors came not for a little water beg but for the word |
| 198 | Leraren op scholen waren de afgelopen jaren oneens over de orthografie van het woordje | 25 | Teachers on schools were the past years in disagreement about the orthography of the word |
| 199 | Alle gebieden die de jongeren nog gaan bezoeken waren gemarkeerd gebruikmakende van het woordje | 26 | All areas that the youngsters still went visit were marked using the word |
| 200 | Vannacht had een ongeluk plaatsgevonden omdat de chauffeur luisterde naar een radioshow over het woordje | 27 | Last night had an accident taken place because the driver listened to a radio show about the word |
